# Barriers and Facilitators of Partner Treatment of Chlamydia: A Qualitative Investigation with Prescribers and Community Pharmacists

**DOI:** 10.3390/pharmacy6010017

**Published:** 2018-02-08

**Authors:** Helen Wood, Caroline Hall, Emma Ioppolo, Renée Ioppolo, Ella Scacchia, Rhonda Clifford, Sajni Gudka

**Affiliations:** Division of Pharmacy, School of Allied Health, The University of Western Australia, Perth 6009, Australia; helen.wood@uwa.edu.au (H.W.); 21132567@student.uwa.edu.au (C.H.); 21114143@student.uwa.edu.au (E.I.); 21119548@student.uwa.edu.au (R.I.); 21312286@student.uwa.edu.au (E.S.); rhonda.clifford@uwa.edu.au (R.C.)

**Keywords:** chlamydia, pharmacy, pharmacist, contact tracing, partner therapy, accelerated partner therapy, expedited partner therapy, patient-delivered partner therapy, barrier, facilitator

## Abstract

Chlamydia trachomatis is the most frequently-notified sexually transmitted infection in Australia. Effective and timely partner treatment of chlamydia is essential to reduce overall prevalence and the burden of infection. Currently in most of Australia, the only avenue for partner treatment of chlamydia (“standard partner therapy”) is a tedious, and often inconvenient, process. The barriers and facilitators of standard partner therapy, and newer models of accelerated partner therapy (APT), need to be identified in the Australian setting. Additionally, the potential role of community pharmacists need to be explored. Semi-structured interview guides for two key stakeholder groups (prescribers and pharmacists) were developed and piloted. Eleven prescribers (general practitioners, sexual health clinicians and nurse practitioners) and twelve pharmacists practicing in the Perth metropolitan region were interviewed. Key reported barriers to standard partner therapy were lack of or delayed chlamydia testing. Key facilitators included ability to test and educate sexual partner. Key barriers for APT included prescribers’ legal responsibility and potential for medication-related adverse effects. Healthcare provider consultation and chlamydia testing were seen as potential facilitators of APT. Pharmacists were receptive to the idea of expanding their role in chlamydia treatment, however, barriers to privacy must be overcome in order to be acceptable to prescribers and pharmacists.

## 1. Introduction

Chlamydia trachomatis (hereafter referred to as chlamydia) is the most frequently notified sexually transmitted infection (STI) in Australia [1]. In Western Australia, the incidence of chlamydia has risen by 193% in the last decade, with 11,807 new cases reported in 2016 alone [1]. The asymptomatic nature of chlamydial infections in up to 70–90% of women and over 50% of men leaves the control of infections challenging [2]. If left untreated, chlamydial infections may lead to a number of severe, and possibly permanent, sequelae [3]. Complications include urethritis and epididymitis in men, and pelvic inflammatory disease, ectopic pregnancy and infertility in women [3,4,5]. Effective and timely treatment is, therefore, imperative in both reducing the likelihood and severity of sequelae, and in controlling the spread of chlamydia.

**Figure 1:**
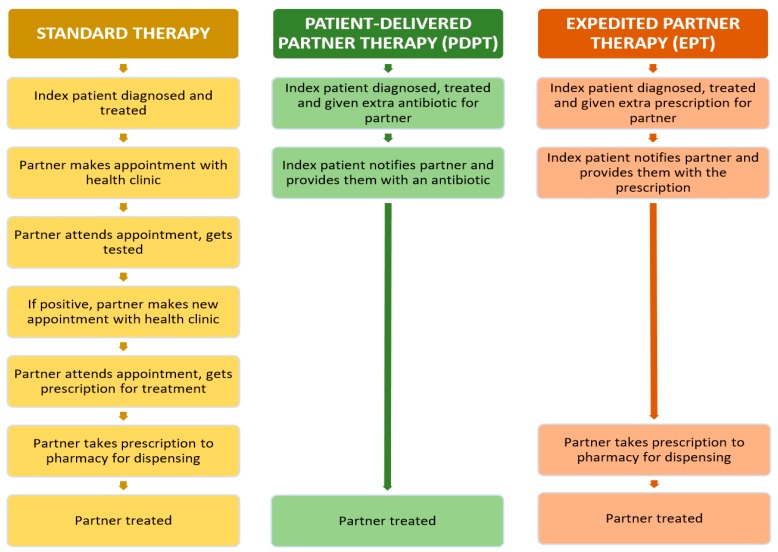
The current processes for standard partner therapy, PDPT and EPT. Many steps involved in standard partner therapy (and, thus, the barriers associated with these steps) are removed in the simpler models of PDPT and EPT. Figure is adapted from one published by Estcourt et al. [16].

The high prevalence of chlamydia is partly attributed to inadequate or incomplete management of the initial “index” patients’ sexual partners. If chlamydia-positive, partners can spread infection to subsequent partners or re-infect a treated index patient [6,7]. Currently in Western Australia, the only way to access partner treatment of chlamydia (“standard partner therapy”) is a tedious, and often inconvenient, process (Figure 1) [8,9,10,11]. Standard partner therapy may pose several barriers to sexual partners, including cost, time and lack of accessibility [12,13,14,15]. These barriers may increase chlamydia transmission by preventing effective and timely treatment of partners. In an effort to overcome these key barriers, novel forms of partner therapy have been trialled or implemented in Sweden, the United States (US), Canada, the United Kingdom and Australia, to complement—rather than replace—standard partner therapy. In this paper, Accelerated Partner Therapy (APT) is used as a blanket term to describe two common alternative methods of partner treatment for chlamydia, that reduce the time for partners to be treated. We refer to these two separate models as Patient-Delivered Partner Therapy (PDPT) and Expedited Partner Therapy (EPT), shown in Figure 1.

Both alternative models rely on the recently treated index patient to directly deliver a treatment option to their sexual partner(s). In Patient-Delivered Partner Therapy (PDPT), the prescriber provides an extra dose of azithromycin to the index patient to treat their sexual partner [17]. In Expedited Partner Therapy (EPT), the index patient delivers a prescription to the partner, which they can take to a community pharmacy for dispensing [17]. The need for sexual partners to attend a health clinician appointment, conduct a self-collected chlamydia test and wait for a positive diagnosis is removed prior to treatment in both of these innovative models. International qualitative consultations have shown that APT overcomes frequently cited barriers experienced by the standard partner therapy model; cost of appointments and testing, inconvenience of attending appointments and poorly accessible healthcare services [18,19,20,21,22]. With these barriers removed, treatment becomes more accessible to a wider range of sexual partners.

APT is formally utilised in some international and Australian healthcare settings. It has been implemented in Sweden, much of the US, and two Australian states [17,19,23]. After careful consideration of the international studies demonstrating efficacy, recent amendments to the Victorian and Northern Territory state legislation now allow the practice of both PDPT and EPT, in accordance with established clinical guidelines [17,23]. However, the guidelines were not written with consideration of any Australian data or research [17,23]. Additionally, there have been no post-implementation studies or analysis of these guidelines used in Australia. Hence, we do not know the reach, usability, effectiveness and acceptability in the Australian setting. We also do not know if barriers to PDPT or EPT that have been identified internationally are relevant in our unique healthcare and geographical settings. Australia’s healthcare system is a combination of free or partly subsidized public care (Medicare), and privately funded care (through optional private healthcare insurance) [24]. Geographically, Western Australia residents are relatively isolated, with the capital city (Perth) being regarded as one of the most isolated cities in the world [25]. Furthermore, many residents live in regional and remote areas where access to primary health care can be challenging due to distance or lack of service [26]. 

The health professionals involved in standard partner therapy in Western Australia include general practitioners (GPs), sexual health clinicians and nurse practitioners (NPs), collectively referred to in this article as “prescribers”. If a model of EPT was introduced, community pharmacists would also assume a central role in chlamydia treatment for sexual partners. As such, it is important to understand the views and attitudes of these key stakeholder groups. Thus, the objectives of this research were to: (1) investigate barriers and facilitators to standard partner therapy for chlamydia in the Perth metropolitan region, as reported by prescribers; (2) investigate barriers and suggested facilitators to APT in the Perth metropolitan region, as reported by prescribers and community pharmacists; and (3) to explore the potential role of community pharmacists in partner therapy of chlamydia. We intend that the findings from this study will inform an effective alternative pathway for partner treatment of chlamydia and, thus, may reduce reinfection rates and complications arising from untreated chlamydial infections.

## 2. Materials and Methods 

### 2.1. Ethical Considerations

Ethics approval for this study was obtained from the University of Western Australia Human Research Ethics Committee (HREC RA/4/1/9092) on 24 August 2017. All participants were provided with participant information and written informed consent was obtained, in accordance with approval requirements. 

### 2.2. Study Design

The study was conducted using a qualitative, exploratory design. Semi-structured, face-to-face interviews were conducted with prescribers and community pharmacists practicing in the Perth metropolitan region. The interviews explored the health professionals’ views on standard and accelerated chlamydia therapy models for partners. Interviews were divided into two groups (prescribers and pharmacists), with a single research member conducting all interviews within a given participant group to ensure interviewer consistency. Each interviewer was provided with open-ended questions, prompts, flow-charts for defining each therapy model, participant information forms and participant consent forms. Interviews ran for approximately 15–30 min each, and were conducted until saturation of ideas was reached (no new themes emerged during interviews). Participants each received a retail gift voucher valued at AU$50 in acknowledgement of their contributions.

### 2.3. Interview Design

Interview questions were developed by the research team after a review of current literature, and were used to guide interviewers, and provide consistency between interviews. Probe questions were also developed to further explore participant responses, if and when necessary. Key topics discussed amongst prescribers included: usual practice for partner notification, effectiveness of standard partner therapy, concerns and benefits of APT, suggestions to improve partner therapy, and pharmacists’ potential role in chlamydia therapy of partners. Similarly, key topics discussed amongst pharmacists included: their opinions on the community pharmacists’ potential role in chlamydia management of partners, attitudes towards participating in EPT, and factors to consider in the pharmacy environment for uptake of EPT. The interview questions are available upon request by contacting the corresponding author.

### 2.4. Pilot

Prior to data collection, the interview questions and structure for both participant groups were piloted internally amongst researchers. There were no specific changes made to the questions after this revision, with only two leading probes removed from the prescriber interviews. The interview structures were reviewed again by researchers after completion of the first three interviews within each participant group to ensure questions were comprehensible to participants and met research objectives. No changes were made to the interview questions or structure at this point.

### 2.5. Participant Recruitment

Prescribers and community pharmacists were recruited in August 2017 on a voluntary basis. Prescribers were recruited from medical practices with special interests in sexual health (as identified on the Healthshare website), sexual health clinics (as identified on the Australian Government Department of Health website) and the Australian College of Nurse Practitioners. Health clinics selected by purposeful sampling were contacted via telephone, and then sent email invitations for prescribers to participate in the study (n = 20). An email invitation was sent to the Western Australian representative of the Australian College of Nurse Practitioners for dissemination to eligible NPs. Four participants were recruited through medical practices with special interests in sexual health; six participants were recruited through sexual health clinics; and one participant was recruited through the Australian College of Nurse Practitioners.

The 2017 Pharmacy Registration Board of Western Australia was used for recruitment of pharmacists from community pharmacies listed in the Perth metropolitan region (n = 460). Systematic sampling was used, with every fourth pharmacy contacted via telephone (n = 115). Five of the contacted pharmacies were not interested in participating. The remaining pharmacies were emailed invitations to participate in the study (n = 110). Due to initial low response rates, additional pharmacists were subsequently recruited via social media advertising on two closed pharmacist Facebook groups, and through professional networks of researchers.

### 2.6. Inclusion Criteria

Prescribers and pharmacists were included in the study if they:

Prescribers
  - Were registered and practicing as either a GP, sexual health physician, or NP in the Perth metropolitan region;
  - Had treated at least one index patient diagnosed with chlamydia in the past three months; and
  - Were able to speak and read English
Pharmacists
  - Were registered as a pharmacist in the Perth metropolitan region;
  - Worked a minimum of 20 h per week in a community pharmacy; and
  - Were able to speak and read English

### 2.7. Exclusion Criteria

Prescribers and pharmacists were excluded from the study if they:

Prescribers
  - Worked in a clinic exclusively treating groups at high risk of complicated chlamydial infections and/or other concurrent STIs (e.g., men who have sex with men or Aboriginal and Torres Strait Islander people)
Pharmacists
  - Exclusively worked in a hospital pharmacy

### 2.8. Data Collection

The semi-structured interviews were held between 29 August and 20 September 2017. Data was collected in three ways from the interview sessions:

1. Audio recordings (audiotaped on two separate devices)
2. Researcher field notes
3. Participant demographics form

### 2.9. Data Entry and Analysis

The recordings of each interview were transcribed verbatim by the research team. All transcripts were cross-checked between research team members to ensure accuracy and consistency of data entry. Transcripts were then thematically analysed independently by four researchers to ensure methodological rigour. An inductive approach was used, as the research objectives guided the data analysis [27]. The process of thematic analysis involved was guided by Ritchie and Spencer [28]:

1. Immersion in data: reading and re-reading transcripts.
2. Developing a first impression: highlighting key words and phrases that captured major thoughts.
3. Developing a coding scheme: labelling similar codes that accurately depict the data. A code was a raw data point that was grouped with similar data points to form a theme.
4. Identification of themes: establishing themes and super-themes based on relationships between categories. Theming was done using pen and paper, and similar barriers and facilitators were grouped together. A theme name was created for each group.
5. Cross-checking between researchers: checking raw data and ensuring accurate representations of results. Where discrepancies existed, the raw data were examined to view responses in context and a consensus was reached.

### 2.10. Definitions

For the purpose of this research, we defined a barrier as “a factor that prevents or hinders the process of treating sexual partners for chlamydia” and a facilitator as “a factor that may promote or accelerate the process of treating sexual partners for chlamydia”. These definitions were adapted from existing literature during the literature review process undertaken by research members.

## 3. Results

A total of 23 semi-structured interviews were conducted with 11 prescribers and 12 community pharmacists. Table 1 describes the demographics of the interviewed participants.

Interviewed prescribers said they each requested between 10–100 chlamydia tests per month; approximately 10% of those were chlamydia-positive. Prescribers reported treating chlamydia-positive index patients, and encouraging the index patients to notify their own sexual partner(s)—a process known as “partner notification”. Occasionally, prescribers would carry the partner notification process out themselves.

### 3.1. Standard Partner Therapy 

Upon analysing data from prescriber interviews, several barriers and facilitators relating to the standard partner therapy were discussed. The themes are detailed in Table 2 with key illustrative quotes from participants:

The majority of prescribers indicated that they do not always follow the standard partner therapy pathway outlined in Figure 1. Instead, they adapt a blend of standard partner therapy (by seeing the sexual partner) and APT (by providing treatment before diagnosis has been made):

“I’d say probably 90% or more [of partners] agree to just have the treatment that [testing] day, because it saves them coming back for a second visit”—Prescriber 2

“We’re not legally supposed to provide a script for azithromycin for the partner…or give the patient a dose of azithromycin to take home for their partner…but occasionally we do do that here”—Prescriber 6

“Usually we would offer the partner treatment, regardless of whether their test is positive or negative, because we work on giving people empirical treatment”—Prescriber 8

### 3.2. Accelerated Partner Therapy

Upon analysis of the data, several themes arose relating to APT. These themes were: prescriber issues; pharmacy issues; medication issues, and process issues. Prescriber issues were the barriers and facilitators that were relevant at the time of prescribing additional partner treatment, whether in medication or prescription form. Pharmacy issues were the points raised that regarded pharmacy-based features or processes. Medication issues were the points raised by participants as to the use of azithromycin in each model of APT. Process issues were the barriers and facilitators to the overall concepts of PDPT or EPT. The themes and subthemes are detailed in Table 3 with key illustrative quotes from participants:

### 3.3. Pharmacists’ Role and Pharmacy Services

Some prescribers made the comparison between the pharmacists’ hypothetical role in EPT and emergency hormonal contraception (EHC), referred to in this quote as the “morning after pill”:

“Having a pharmacist dispense the morning after pill is no different from them dispensing azithromycin as a stat dose for somebody with suspected chlamydia”—Prescriber 5

When questioned on their current role in standard partner therapy, all pharmacists described their current position in terms of education, counselling, health promotion, and/or prevention of STIs.

“Giving information about the disease state or how it is spread…encourage people to have check-ups with their doctor…We can also talk to them [about] how to take medication…following up on partners.”—Pharmacist 2

“To help provide medications, to counsel patients…I think preventive health as well, so like public health and public awareness”—Pharmacist 4

Upon introducing the concept of EPT to pharmacists, many made comparisons between EPT and similar services already provided in community pharmacies. These services included the provision of one-off medications such as EHC, and antiviral medication for herpes labialis (cold sores). In light of this, they felt their role would not significantly change if EPT were to be implemented, and could appreciate the benefit of presumptively treating sexual partners with azithromycin:

“It’s benefit and risk at the end of it…If you use [azithromycin] and you don’t actually have [chlamydia], you wouldn’t really have any significant risk.”—Pharmacist 6

All pharmacists and most prescribers perceived community pharmacists as being capable of fulfilling this potential role in EPT.

## 4. Discussion

This qualitative study is the first of its kind to explore the barriers and facilitators to both standard and accelerated models of partner treatment of chlamydia, as reported by prescribers and community pharmacists. It is also the first study to seek the perspective of Australian community pharmacists with respect to EPT. Many of our findings were consistent with those in existing literature, though there were some new insights discovered.

### 4.1. Standard Partner Therapy

The key barriers and facilitators to the standard partner therapy model (as shown in Figure 1) identified in this study were relatively consistent with existing national and international literature which have explored GP and NP views on chlamydia testing [29,30,31,32]. A major barrier faced by prescribers is sexual partners’ deprioritizing of the need for testing and, therefore, they remain untreated. While facilitators to standard partner therapy exist, APT is intended to be most beneficial for the subgroup of sexual partners who do not place high priority on seeking chlamydia testing and treatment. We did not identify any prescriber embarrassment with discussing chlamydia or sexual health that has been reported elsewhere [33], but this may be attributed to our purposive sampling of prescribers with particular interest in sexual health who have recently diagnosed at least one index patient with chlamydia. 

### 4.2. Accelerated Partner Therapy (APT)

#### 4.2.1. Prescriber Issues

The findings from this study are consistent with the existing literature, with medico-legal concerns and fears of liability being frequently reported by prescribers as barriers in both national and international papers [22,32,34,35]. While the lack of opportunity to interact with the sexual partner was often identified as a barrier to APT, some participants recognized that the missed opportunities for counselling and sexual health education could be overcome by the inclusion of community pharmacists in the hypothetical model. Participants suggested that, with appropriate training, pharmacists could, and were, willing to assume a more central role in partner treatment of chlamydia. Other implemented models of APT have also overcome this barrier with prescriber- or pharmacist-provided written information packs, to educate sexual partners and encourage further testing [17,20,23].

#### 4.2.2. Pharmacy Issues

The lack of privacy offered in the pharmacy retail environment was highlighted several times as a barrier for EPT. Despite this, most pharmacists agreed that the private consultation rooms in their pharmacy provided adequate privacy for an EPT consultation. It is well-documented that privacy and confidentiality are essential components of any form of partner treatment model [21,36,37,38]. Post-implementation research into pharmacist-provided EHC in Australia has shown that privacy is not always assured during sexual health consultations. Mystery shopping was utilized in two studies; 100 community pharmacies participated in the first study, and 23 participated in the second [39,40]. In the first study the authors considered privacy to be adequate in 90% of consultations, when pharmacists directed the mystery shopper away from the counter and other customers [39]. In the second study, 48% of consultations met these criteria for privacy [40]. Additionally, a postal survey of 427 Australian community pharmacists was conducted four years after the introduction of pharmacist-supplied EHC; 59.5% of participants preferred to conduct EHC consultations in a “part of the pharmacy where confidentiality could be assured” or an area free of other customers [41]. The authors did not describe how confidentiality was assured if other customers were present, nor did they identify reasons why 30.5% of participants responded that they did not ensure customers’ privacy needs were met. Further exploration of this issue may be required.

Some pharmacists indicated the lack of time to use private consultation rooms was a barrier. These findings emphasise the importance of having networks of staff available to support pharmacists if they were to assume a role in managing partner treatment via EPT. 

#### 4.2.3. Medication Issues

A frequently-cited barrier to APT was the potential for medication-related adverse events. However, the majority of pharmacists—and a few prescribers—acknowledged that azithromycin for chlamydia treatment is a single dose, with few side effects and low potential for allergic reactions or drug interactions. Pharmacists stated they would feel comfortable providing azithromycin to sexual partners who had not yet been diagnosed with chlamydia, as they believed the benefits would outweigh the risks.

In terms of medication safety, previous studies have acknowledged that there was no significant increase in serious adverse drug reactions due to the use of azithromycin for sexual partners who receive the medication through APT [4,35,42,43]. 

Several prescribers were concerned about the potential for antibiotic resistance to azithromycin by empirically treating sexual partners. In light of this, it was surprising that most of the prescribers interviewed already provided presumptive antibiotic treatment for sexual partners at least some of the time, rather than waiting for pathology results. While we anticipated that some prescribers may adopt this practice, based on other Australian research, we did not expect empirical treatment to be offered by almost every prescriber [32]. This may suggest that prescribers’ concern regarding antibiotic resistance when using APT is not a prohibitive barrier to their acceptance, as they are already comfortable with prescribing azithromycin prior to a diagnosis. Furthermore, the risk of antibiotic resistance arising from a single dose of azithromycin has been found to be negligible, with Khan et al. stating that the risk is no greater than if sexual partners followed the standard partner therapy pathway to receive treatment [44]. Khan et al. commented that, by improving accessibility to chlamydia treatment, chlamydia prevalence will decrease in the general population and, thus, an overall reduction in antimicrobial use will be observed [44].

Some prescribers stated they would be comfortable having a telephone- or app-based consultation with the sexual partner before leaving a prescription at reception. A telephone-based model of partner treatment, similar to what was described by prescribers in our study, was trialled by Estcourt et al. in 2011; called APTHotline, the notified sexual partner called a hotline number and had a telephone consultation with a health adviser, senior nurse or doctor [16]. The index patient or sexual partner were then able to collect an APT pack from the clinic’s reception. The pack contained azithromycin and relevant information, a testing kit for *Chlamydia trachomatis* (chlamydia) and *Neisseria gonorrhoeae* (gonorrhoea), and condoms [16]. Estcourt et al. found that APTHotline improved both partner treatment rates and time taken for partner to access treatment, and was widely acceptable to index patients and sexual partners [16].

#### 4.2.4. Process Issues

One barrier that was identified by some participants related to the potential for consumers to become desensitized to safe sexual measures if treatment became too accessible. To our knowledge, this barrier has not been described in previous studies. While it is a concerning thought, there is no evidence to suggest that this has occurred in other countries where APT is implemented. Additionally, similarities can be drawn between accessing chlamydia treatment and EHC from a pharmacist, as both scenarios involve at least one instance of unsafe sexual intercourse. When reviewing the evidence, the Bixby Centre for Global Reproductive Health concluded that there is overwhelming evidence that provision of EHC does not increase sexual risk-taking behaviour, even when it is provided in advance of need [45]. Rather, increasing access to EHC increases the likelihood of its use [45]. It is, therefore, possible that by making azithromycin more easily accessible for sexual partners, it is more likely to be used by those who need it. 

The provision of chlamydia self-test kits was discussed as a possible facilitator to APT, which could encourage testing and instigate a conversation about the potential for co-infection of other STIs. Community pharmacies were suggested as a location for this service, so partners could opt for chlamydia testing when they access treatment. Accessible chlamydia testing through community pharmacies has been studied previously in an Australian context [46,47]. The key finding from both studies was that pharmacy-accessible chlamydia testing was feasible, convenient and acceptable to pharmacists and consumers [46,47]. 

Overall, we observed that the barriers for standard partner therapy could be overcome through the implementation of APT. Additionally, the facilitators for standard partner therapy (the ability to assess, test and educate sexual partners) could be key features of APT by taking participants’ suggested facilitators into consideration when writing clinical guidelines. 

### 4.3. Role of the Pharmacist

There is a general movement within the pharmacy profession where the scope of the pharmacists’ role is expanding [36,48]. Upon introducing the concept of EPT, pharmacists in this study were open to further developing their role in chlamydia management. This finding is consistent with two international studies, where an “overwhelmingly positive response” was seen by pharmacists towards their increased involvement in chlamydia treatment [48]. This positive response is crucial, as the willingness of community pharmacists to take part in EPT is one of the first steps towards trialling such models in practice. Likewise, many prescribers were receptive to the notion of APT; however, similarly to existing literature, several revealed they would feel more comfortable with the models if they had some form of verbal contact with the partner [49,50]. This may be a reason why several prescribers in this study were more inclined to use EPT, rather than PDPT, as partners have contact with a health professional (i.e., pharmacist) in the EPT model.

### 4.4. Strengths and Limitations

This study had a number of strengths. We recruited key stakeholders (i.e., community pharmacists and prescribers) who offered relevant perspectives on both standard and accelerated therapy models. Furthermore, this study used purposive sampling to recruit prescribers from a diverse range of practice settings. This approach allowed us to gain insight into real-life barriers and facilitators, which may increase the applicability of our research. Understanding these contextual barriers and facilitators provides the opportunity for these barriers to be addressed more effectively, should APT be trialled in Western Australia. This is significant, as the majority of research to date has been conducted in the United Kingdom and the United States of America [19,22,35,36,48,49,50,51] and, thus, may not be entirely applicable to the Australian context.

Our study encountered several limitations. As with all qualitative research, the personal values, assumptions and preconceived ideas of researchers may have subconsciously influenced the interview process, as well as the theming and coding of data. Despite this, effort was made to ensure objectivity in both data collection and analysis. This included the use of verbatim transcripts of the semi-structured interviews, and thematic analyses conducted independently by four members of the research team. We also acknowledge the potential for responder bias from participants in this study. Furthermore, all participation was voluntary; thus, self-selection bias may be a limitation of this study. Lastly, there may also be unrecognised barriers and facilitators to chlamydia treatment of partners that were not uncovered through the use of semi-structured interviews.

### 4.5. Future Direction

Future research is needed on the perceived barriers and facilitators to both standard and accelerated partner therapy, as reported by index patients and their sexual partners. Involving such relevant stakeholders is likely to provide additional insights into the true acceptability and uptake of APT in a Western Australian healthcare settings. Furthermore, conducting similar research with prescribers and community pharmacists in rural and remote Western Australia may further increase the applicability of APT in this setting. Additionally, a deeper understanding of privacy in a community pharmacy setting is required, not just from a wider pool of pharmacists, but also from the perspective of sexual partners.

For a successful pilot of APT models in the Perth metropolitan region, relevant barriers must firstly be addressed and interventions tailored to the setting of use, in order to evaluate overall feasibility and applicability. Furthermore, it is apparent that some of the reported barriers for APT are simply logistical in nature, which may be overcome through the development and introduction of clinical guidelines, funding and legislative changes supporting APT in Western Australia. By overcoming the barriers to APT, we speculate that chlamydia treatment rates amongst partners may increase. This, in turn, may reduce the overall prevalence and burden of chlamydia.

## 5. Conclusions

Treating partners of chlamydia-positive index patients is important in order to break the cycle of infection. The results of this study provide insights into both the current and potential barriers and facilitators of chlamydia treatment of partners, as well as the potential role pharmacists could have in partner treatment of chlamydia. 

Prescribers recognised existing barriers to the standard therapy model, which may be overcome through the use of APT. Offering APT as an alternative to the standard therapy model may, therefore, provide an option for partners who are otherwise unwilling or unable to seek treatment the usual way. Several opportunities for community pharmacists to participate in partner treatment were identified. Given their accessibility, current scope of practice, and acceptance of EPT, it is possible that community pharmacists could assume a central role in treating sexual partners of chlamydia-positive index patients. 

This study was the first to investigate the perspectives of prescribers and pharmacists regarding partner treatment of chlamydia in the Perth metropolitan region. To our knowledge it is the first study to identify pharmacist-perceived barriers and facilitators to EPT in an Australian setting. Overall, the findings of this study highlight the complexity of the factors that may need to be considered when treating sexual partners for chlamydia in the Perth metropolitan region, and it is impossible for one model of care to adequately address the needs of every partner.

## Figures and Tables

**Table 1:** Demographic characteristics of study participants.

	Prescribers (n = 11)	Pharmacists (n = 12)
Median age in years (range)	45 (31–69)	30.5 (27–52)
Median years of experience in current role (range)	14 (1–30)	8 (5–18)
Gender F (%)	9 (82%)	8 (67%)
Job title * (n)		
Community pharmacist	N/A	12
General practitioner	4	N/A
Medical director	1	N/A
Medical officer	1	N/A
Nurse practitioner	2	N/A
Public health physician	2	N/A
Sexual health physician	5	N/A
Median hours worked per week (range)	(not measured)	38 (20–60)

* Some prescribers have more than 1 job title.

**Table 2:** Barriers and facilitators to standard partner therapy, as described by prescribers (n = 11).

Standard Partner Therapy		Key Illustrative Quote
**Barriers**	Sexual partner does not seek testing	“For casual contacts I think we probably don’t have a very good hit rate because it’s hard for them to get partners in…and it is time consuming for the contacts to come and get tested.”—Prescriber 1 “But, you know, we don’t live in an ideal world, and often people will not present for screening or for testing”—Prescriber 9
	Delay to testing	“There may be a delay, so [the index patient] may get re-infected. There may be a delay in getting appointments, they might not understand the importance of it.”—Prescriber 7
**Facilitators**	Able to test for other sites of infection	“There’s advantages of [the sexual partner] coming in to get tested because you can erase a dramatic infection—for example, rectal infection and that is treated differently”—Prescriber 1
	Able to assess sexual partner	“We’re able to see them and get a history and assess for any risks outside of chlamydia. See what their medication tolerance and compliance would be.”—Prescriber 3
	Able to provide education	“We’ll educate them on what the treatment is, what the treatment options are.”—Prescriber 4

**Table 3:** Barriers and suggested facilitators to Accelerated Partner Therapy, as described by prescribers (n = 11) and pharmacists (n = 12).

Prescriber Issues		Key Illustrative Quote
**Barriers**	Legal professional responsibility	“It’s a safe drug but it’s my name on the box”—Prescriber 7
	Prescribing for an unseen partner ▪ Lack of opportunity for consultation and education ▪ Empirical treatment	“My biggest worry is that if we don’t [test and educate partner] then we’re not actually giving people information that they might need about protecting themselves”—Prescriber 6
	Lack of remuneration for service	“The reality is unfortunately that unless there’s a Medicare billing item attached to it, it can be a barrier for [prescribers] to do it.”—Prescriber 10
**Suggested Facilitators**	Discreet prescription annotation to indicate need for extended consultation	“There could be some sort of standard note that we attach [to the prescription] to say that this person hasn’t been seen, could you screen for allergies … I’d feel very comfortable doing that”—Prescriber 6 “If [the prescription] is annotated in quite a subtle way, but quite clear to the health professional that would be adequate. So it’s not obvious to anyone”—Pharmacist 12
	Pharmacist training	“It is a field that pharmacists can be educated on so it’s not something that we just can’t do”—Pharmacist 8
	Medicare subsidisation	“To have some sort of Medicare billable item would be good because a lot of [prescriber] work goes unbilled”—Prescriber 10
**Pharmacy Issues**		
**Barriers**	Lack of pharmacy staff	“The consult room is often there but it’s hard to use…you often don’t have time to do that if you’re the only pharmacist working”—Pharmacist 3
	Lack of privacy in pharmacy	“[Pharmacists] ask at the top of their voice what you’re here for, have you taken this before, which is all part of their job but then sometimes it’s quite personal information that they ask”—Prescriber 6
**Suggested Facilitators**	Offer of a confidential consultation	“[In the consultation room] we often spend much more than five minutes with clients who are interested in knowing more about their medications, knowing more of the treatment options”—Pharmacist 8
	Financial remuneration	“If it’s more of an extended consultation I think that could possibly come under Medicare payment for a service if it was something that when you sat down, you had to explain what chlamydia is, what the treatment is about … I think it’s fair to offer remuneration for that”—Pharmacist 2
**Medication Issues**		
**Barriers**	Potential for adverse drug reactions	“There is the safety aspect obviously because you don’t know what that person’s medical history is, you don’t know what allergies they have, what other medication they’re on … you don’t want to influence somebody in another way without even knowing them”—Prescriber 5
	Potential for antibiotic resistance	“[The] concerns are mainly [that] you don’t actually know if the partner actually has the STD, so resistance comes to mind”—Pharmacist 3
**Suggested Facilitators**	Prescriber-led telephone consultation with partner prior to writing prescription	“It might be worthwhile if we could just get the partner on the telephone and just take a general history”—Prescriber 6
**Process Issues**		
**Barriers**	Too accessible	“If they can access treatment a lot easier, they’re going to be a lot more reckless with their behaviour”—Prescriber 5 “… And they think, it’s easy to treat so it’s not as important to prevent”—Pharmacist 4
	Inability for further testing and follow up care	“You don’t know that [partners] have had an opportunity to…get tested for other STIs, because if you’ve got one, you’re more likely going to have another”—Prescriber 7
**Suggested Facilitators**	Provision of chlamydia self-test kits	“You could have testing kits in the pharmacy that [partners] could potentially pick up … when they’ve picked up their azithromycin”—Prescriber 1
